# Effects of an intensive slow cortical potentials neurofeedback training in female and male adolescents with autism spectrum disorder: Are there sex differences?

**DOI:** 10.1007/s00508-021-01989-7

**Published:** 2021-12-15

**Authors:** Sonja G. Werneck-Rohrer, Theresa M. Lindorfer, Carolin Waleew, Julia Philipp, Karin Prillinger, Lilian Konicar

**Affiliations:** Department of Child and Adolescent Psychiatry. Währinger Gürtel 18–20, Medical University of Vienna, 1090 Vienna, Austria

**Keywords:** Female ASD, Neurofeedback therapy, Comorbid psychopathological symptoms of ASD, Cognitive flexibility, Emotion recognition

## Abstract

**Background:**

This study aims to compare the effects of neurofeedback training on male and female adolescents with autism spectrum disorder (ASD). Furthermore, it examines sex differences regarding improvements in co-occurring psychopathological symptoms, cognitive flexibility and emotion recognition abilities. The study might provide first hints whether there is an influence of sex on treatment outcomes.

**Methods:**

Six female and six male adolescents with ASD were matched according to age, IQ and symptom severity. All participants received 24 sessions of electroencephalography-based neurofeedback training. Before and after the intervention, psychological data for measuring co-occurring psychopathological symptoms as well as behavioral data for measuring cognitive flexibility and emotion recognition abilities were recorded.

**Results:**

Caregivers rated statistically significant higher psychopathological problems in female than in male adolescents with ASD at baseline. Apart from that, no statistically significant sex-related differences were revealed in this sample; however, male adolescents tended to report greater improvements of externalizing, internalizing and total symptoms, whereas females experienced smaller improvements of externalizing and total problems, but no improvements of internalizing problems. Regarding caregivers’ assessments, more improvement of total problems was reported for females. For males, only improvements of internalizing and total problems were described.

**Conclusion:**

This study reveals preliminary results that sex-related differences might play a role when evaluating treatment outcomes after neurofeedback training regarding comorbid psychopathological symptoms. Adolescents’ self-report and parental assessments, especially concerning psychopathological symptoms, should be combined and considered in future studies to help prevent sex bias in adolescents with ASD.

## Introduction

Autism spectrum disorder (ASD) is generally described as a neurodevelopmental disorder characterized by impairments in reciprocal social interaction, a deficit in social communication, repetitive and restrictive behavior [1]. In recent years, the prevalence of ASD has increased due to improved diagnostic criteria and greater awareness of ASD symptoms; however, there is a lack of research on ASD in women, as scientific studies predominantly included male subjects [2, 3]. In females ASD is often unrecognized, undiagnosed or misdiagnosed due to a different phenotype presentation in females and a lack of sex-specific elements in diagnostic procedures [2]. This fact can lead to a shortage of interventions specifically targeting the impairments of females with ASD. There are different theses that defend the hypothesis of a rarer occurrence of ASD in females. One is the female protective effect, which states that women require a higher genetic and/or environmental risk to be affected by it than men, meaning that sex can be a protective factor. Another theory is the extreme male brain theory. This states that the male brain is defined as thinking systematically and having less empathy, whereas the female brain is defined with the opposite characteristics. Therefore, ASD would be an extreme expression of a typically male brain [3]; however, the female camouflage effect is probably one of the most interesting explanatory approaches to understand the underdiagnosis of ASD in females. It posits that females with ASD often try to compensate and hide their social difficulties from others [4, 5]. In general, girls with ASD show a great desire to establish interpersonal relationships, want to fit in and aim to avoid negative judgements and reactions from society [6]. A study by Cola and colleagues [7] showed that after speaking to persons who did not know the diagnosis, the social abilities of girls with ASD were rated more typical than those of boys with ASD. This investigation exemplified that recognizing ASD symptoms is apparently more difficult in girls than in boys [3].

Loomes and colleagues [8] reported that the male to female ratio in ASD is not 4:1 (as assumed in the diagnostic and statistical manual of mental disorders, fifth edition, DSM‑5 [9]) but rather 3:1, as females with ASD are at a disproportional risk of not being diagnosed. This comes partly from diagnostic measures, which barely include female characteristics of ASD [10, 11] or diverging sex-related clinical presentations of ASD. In early childhood for example, more depressive and anxious behavior was seen in girls with ASD and more restricted, repetitive and stereotyped behavior in boys with ASD [12, 13]. Interestingly, while in the categories of circumscribed interests and unusual preoccupations [14, 15] sex-related differences in ASD were found, no differences were found in the total scores of the autism diagnostic interview (ADI‑R [16]) and the ADI‑R subscale restrictive and repetitive behavior.

Therefore, it is crucial to consider that a solely male understanding of ASD lowers the probability of detecting ASD in females [17, 18], leading to belated diagnoses and treatment delay [17–20], yielding sex bias-based problems in health, identity and well-being of females with ASD [21].

### Deficits in executive functions and emotion processing in ASD

Besides information updating and inhibition control, cognitive flexibility is a major part of executive functioning [22] and assumed to be impaired in patients with ASD [23]. Willcut et al. [24] found strong effect sizes for differences in cognitive flexibility between patients with ASD and typically developing subjects; however, meta-analysis yielded controversial results [25]. Thus, cognitive flexibility tasks may be difficult for patients with ASD for other reasons, such as learning through feedback and performance clearly depends on the type of tasks and on the task instructions [23, 26]. Hull et al. [27] further described sex differences in performance on cognitive flexibility tasks depending on the type of task; for example, no differences are shown on the Wisconsin card sorting test, while performance on the Trail Making Test was better in females than in males. A functional magnetic resonance imaging (fMRI) study by Shafritz et al. [28] reported lower activation in the frontal, striatal, and parietal regions of the brain, parallel to seemingly different task solving strategies of typically developing subjects and patients with ASD.

Regarding attention deficit hyperactivity disorder (ADHD), a common comorbidity of ASD, much more research was conducted to investigate sex differences. For example, differences in processing speed and working memory between female and male patients with ADHD were reported [29]. Furthermore, a meta-analysis showed more hyperactive behavior, more deficits in motor response inhibition and cognitive flexibility in boys with ADHD than in girls with ADHD [30]. As the symptomatology of ASD and ADHD often appear together, similar sex differences might be found in ASD in future studies.

Regarding emotional processing, the most common finding is an impairment in facial emotion recognition in individuals with ASD [31, 32]. Studies repeatedly showed that patients with ASD exhibit slower reaction times [33] as well as more incorrect classifications of emotion assignments than typically developing subjects [34–36]. Moreover, the gaze at the eyes of others is associated with a higher rate of correct answers in emotion recognition and lower symptom severity [37]. Contrary, atypical brain activations during the presentation of faces with different emotions were associated with deficits in the processing of facial expressions [38]. Regarding sex differences, Kothari et al. [39] found impaired emotional processing in boys with ASD compared to girls with ASD, assuming that girls with ASD probably compensate their underlying deficits.

Here, a general framework is required to determine which cognitive and emotional processes are specifically deficient in males and females in ASD. Furthermore, valid measures linking behavior, psychopathology and neurophysiology need to be established [23].

### Neurofeedback training as a new treatment option in ASD

Currently no standard treatment, such as cognitive behavioral therapy or psychopharmacological interventions target the core symptomatology of ASD or consider sex as a potential influencing factor for treatment outcome [40, 41].

Although biological approaches also do not consider sex as an influencing factor, they aim to change the biological basis of ASD for the purpose of symptom reduction. Regarding the biological basis of ASD, brain imaging studies reported diminished cortical excitability and reduced frontal brain activity related to impaired neural plasticity [42–44]. Parallel, resting state electroencephalography (EEG) studies demonstrated impairments in long-range connectivity between frontal lobe and other cortical regions, together with short-range overconnectivity [45–47]. The most consistent findings regarding abnormal frequency band activity in ASD is an overexpression of slow frequency bands, together with a decreased alpha band power [45, 48].

Therefore, a biological approach targeting exactly these seemingly impaired biological hubs seems to be a promising treatment strategy for ASD. One method enabling subjects to volitionally learn to change brain activity levels is electroencephalography (EEG)-based neurofeedback. In this self-regulation training method, brain waveforms are measured, analyzed and fed back in real time to the subject. Through this feedback (operant conditioning), the participants can learn special stimulus response patterns by altering their brain activity to a desired state. Successful symptom improvements after EEG neurofeedback training were reported in drug-resistant epilepsy [49], ADHD [50–53], criminal psychopathy [54] as well as in migraine [55].

The first case study about neurofeedback training in ASD was conducted by Cowan and Markham in 1994 [56] with an 8‑year-old girl. Using frequency bands as feedback signal, the aim was to suppress the ratio of alpha and theta to beta EEG activity. It could be shown that after 21 neurofeedback sessions there was a decrease of autistic behavior and an increase of sustained attention. The changes were long-lasting as the follow-up after 2 years still showed behavior and attention scores within the normal range. Pineda et al. [57] found neurophysiological improvements, such as increased brain frequency coherence and normalization after neurofeedback training in ASD, whereas Koujizer et al. [58] showed a reduction of theta and delta power in 54% of the participants. In this study, ASD symptoms did not decline but the study participants showed improved cognitive flexibility. This finding is supported by a recent randomized clinical trial [59] showing that the attentional span and the social interaction skills improved in addition to the cognitive flexibility. Van Hoogdalem [60] described that neurofeedback studies in ASD predominantly recruited male participants and the study results were merely transferred to female patients.

Current literature provides evidence that males and females with ASD have different neurophenotypes [2], highlighting the need to explore how sex influences the effectiveness of neurobiologically based treatment modalities for individuals with ASD. To our knowledge, this is the first study looking at sex differences in outcome measures after a neurofeedback therapy in patients with ASD.

## Methods

### Objective and hypotheses

The present study is part of a larger clinical trial investigating neurofeedback therapy for adolescents with ASD, conducted by Konicar et al. [61]. The aim of the main study was to investigate changes in ASD core symptoms and psychopathological concomitant symptoms after slow cortical potential (SCP) neurofeedback therapy in male adolescents with ASD.

The current study aims to extend the findings of this larger clinical trial by conducting the same neurofeedback therapy as in the study mentioned above [61] but in an explanatory manner with female adolescents with ASD. Therefore, the main aim of this current study is to investigate potential sex differences in treatment outcome, i.e. differences regarding psychopathological concomitant symptoms, emotion recognition and cognitive flexibility.

Hence, we expect sex differences in adolescents with ASD regarding the improvements in psychopathological concomitant symptoms (i.e. primary endpoint), cognitive flexibility and emotion recognition (i.e. secondary endpoints) following the EEG neurofeedback training.

### Subjects

Besides the data of the male sample [61], the additional initial female sample consisted of 10 girls with ASD. Due to the coronavirus disease 2019 (COVID-19) pandemic and the associated temporary suspension of scientific projects (in March 2020), the neurofeedback therapy of two female participants had to be discontinued. For the same reason, the planned start of the neurofeedback intervention for two further female subjects had to be cancelled. This resulted in a subsample of six female adolescents with ASD who were matched to six male adolescents with ASD according to age, IQ and the degree of ASD symptom severity. Therefore, a total of 12 right-handed study participants, including 6 female and 6 male adolescents with ASD, based on state-of-the-art diagnostic procedures (ADI‑R [16]; ADOS‑2 [11]) between 12 and 18 years old were included in this study. Participants with an IQ below 70 assessed with the Hamburg-Wechsler intelligence test for children (HAWIK IV [63]), severe head injuries or a severe mental and neurological illness in medical history (i.e., major axis I diagnosis of obsessive-compulsive disorder, psychosis, tics, Tourette syndrome, depression with acute suicidal tendencies, epilepsy) were excluded. Furthermore, study participation with simultaneous participation in other clinical trials or former experience with neurofeedback therapy was not allowed. Accompanying psychopharmacological, behavioral or educational therapy were permitted throughout the study but had to be kept constant during 4 weeks before and until the end of the study. Written informed consent was obtained from all participants and their parents or legal guardians before being enrolled. The study was approved by the Ethics Committee of the Medical University of Vienna in accordance with the Declaration of Helsinki. Subject recruitment and data collection was conducted at the ABC BRAIN LAB, located at the Department of Child and Adolescent Psychiatry at the Medical University of Vienna (Austria).

### Matching

The data of the six female adolescents with ASD were matched to data of six male adolescents with ASD according to age, IQ and symptom severity according to their ASD scores via the total score of the social responsiveness score (SRS) prior to the intervention. Both groups of adolescents (male/female) received the same amount of neurofeedback training sessions and conducted exactly the same study procedure (pre/post measurements).

### Experimental design and SCP neurofeedback training procedure

Premeasurements were conducted within 2 weeks before the neurofeedback intervention started. The participants received neurofeedback training twice a week over the course of 3 months for a total of 24 sessions. The post-surveys were conducted in the same manner as the pre-surveys. The therapeutic neurofeedback procedure is described in detail in Konicar et al. [61]. Brain activity was presented as a graphical object (e.g. fish) on the participants’ screen and reinforced in accordance with prevailing learning theories [62]. One single trial lasted for 10 s: 2 s of baseline recording was followed by an 8 s active regulation phase. In total, 120 neurofeedback trials divided into 3 training blocks, were performed during each training session [61]. The neurofeedback training was divided into 2 training phases, each consisting of 12 sessions and a training break in between.

### Materials

#### Psychological measures: co-occurring psychopathological symptoms

To capture concomitant psychopathological symptoms, a psychological self-report questionnaire, the German version of the youth self-report (YSR/11-18R [23]), was used as well as a parental version (child behavior checklist 6–18 revised; CBCL/6–18R, [23]). Both questionnaires comprised an internalizing problems subscale, an externalizing problems subscale, as well as a total score.

#### Behavioral measures: emotion recognition and cognitive flexibility

A. Measure of emotion recognition (via the Frankfurter Test und Training des Erkennens von fazialem Affekt, FEFA‑2 [64]).

This computerized test reflects the ability to recognize six basic emotions (happiness, sadness, fear, anger, disgust, surprise) and neutral facial expression [65]. The task consists of 50 black and white photographs of adult faces (that differ in age, sex and ethnicity), which should be assigned to the correct emotion. Percentages of correct answers are recorded and analyzed.

B. Measure of cognitive flexibility via tests of attentional performance (TAP 2.3, [66]).

The capacity of cognitive flexibility as one aspect of executive functioning was measured with the subtest flexibility-condition verbal, a set-shifting task of the computer-based test battery TAP-version 2.3 designed for the assessment of various basic attention functions [66]. In this task, a letter and a number appear simultaneously to the left and right side of a fixation point on the screen. The participants are asked to respond alternately to the position of the target stimulus (the number or the letter) by pressing one of the two buttons corresponding to the respective position on the screen (left or right). Reaction time and error rates are recorded and analyzed.

### Statistical analysis

Statistical analyses were performed using IBM SPSS Statistics (Version 27) for Windows. To examine sex differences regarding changes in psychological measures (self-report questionnaire YSR/11-18R; caregiver-report questionnaire CBCL/6-18R), and behavioral measures (emotion recognition measured via FEFA‑2; cognitive flexibility measured via TAP 2.3) across experimental groups, Welch’s t‑tests were used because of the unbalanced study design. The significance level of hypothesis testing was 5%.

## Results

### Subject matching

No significant differences were found between the male versus the female sample of adolescents with ASD comparing age, IQ and SRS total score, as presented in Table 1.

**Table 1:** Matching characteristics at premeasurements

	Male (*n* = 6)^a^	Female (*n* = 6)^a^	Welch’s statistics	
Variable	M (SD)	M (SD)	t (df)	*p*-value
Age (years)	14.33 (1.51)	14.83 (2.04)	0.48 (9.198)	0.640
IQ	104.00 (13.80)	101.60 (25.01)	−0.19 (6.218)	0.857
SRS total score	91.33 (24.60)	96.17 (19.36)	0.38 (9.478)	0.714

Results were considered as significant at an alpha level of *p* < 0.05

*M* mean, *SD* standard deviation, *IQ* intelligence quotient, *SRS* social responsive scale, *df* degrees of freedom

^a^For the IQ data from *n* = 5 participants were included in statistical matching analysis

### Sex differences in premeasures

Analyses revealed significant sex differences in co-occurring psychopathological symptoms, assessed by the caregivers (CBCL) of the adolescents with ASD, before the intervention.

No group differences in female and male subjects were found regarding emotion recognition (FEFA-2), cognitive flexibility (TAP, set shifting task) or self-reported co-occurring psychopathological symptoms (YSR), as shown in Table 2.

**Table 2:** Subjective rating scales and behavioral measures at premeasurements

	Male (*n* = 6)	Female (*n* = 6)	Welch’s statistics	
Parameters	M (SD)	M (SD)	t (df)	*p*-value
Total CBCL score	58.33 (14.26)	78.67 (5.47)	3.26 (6.437)	0.016 ^a^
Total YSR score ^b^	72.17 (24.00)	65.80 (23.68)	−0.44 (8.685)	0.670
FEFA‑2	0.79 (0.05)	0.77 (0.08)	−0.51 (8.717)	0.622
TAP reaction time	765.84 (119.00)	769.78 (110.31)	0.06 (9.944)	0.954
TAP error rate	7.3 (4.08)	5.5 (3.15)	−0.87 (9.391)	0.405

*M* means, *SD* standard deviation, *CBCL* child behavior checklist, *YSR* youth self report, *FEFA* Frankfurter Test und Training des Erkennens von fazialem Affekt, *TAP* test of attentional performance

^a^Results were considered as significant at an alpha level of *p* < 0.05

^b^For the YSR/11-18R, data from *n* = 5 female participants were included in statistical analysis, due to technical problems with YRS implementation

### Sex differences in treatment outcome

#### Behavioral measures: emotion recognition and cognitive flexibility

No sex differences regarding changes in the facial affect recognition test FEFA‑2 [64] were found from before to after neurofeedback intervention, as presented in Table 3.

**Table 3:** Treatment outcome (Δ delta index) of behavioral measures

	Males (*n* = 6)			Females (*n* = 6)			Welch’s statistics	
Variable	Pre-measurement, M (SD)	Post-measurement, M (SD)	Δ	Pre-measurement, M (SD)	Post-measurement, M (SD)	Δ	t (df)	*p*-value
Total FEFA2^a^	0.79 (0.05)	0.81 (0.04)	0.02 (0.02)	0.77 (0.08)	0.79 (0.10)	0.03 (0.08)	0.21 (5.559)	0.841
*TAP 2.3: Flexibility*								
Reaction time	765.84 (119.00)	672.21 (119.63)	−93.63 (98.16)	769.78 (110.31)	649.17 (68.52)	−120.62 (127.11)	−0.41 (9.399)	0.690
Error rate	7.3 (4.08)	5.83 (3.87)	−1.50 (7.40)	5.5 (3.15)	5.17 (4.4)	−0.33 (4.13)	0.34 (7.843)	0.745

*M* Mean, *SD* Standard Deviation, *FEFA* Frankfurter Test und Training des Erkennens von fazialem Affekt, *TAP* test of attentional performance

^a^All values represent percentages (value 1 = 100%)

Furthermore, analyses also showed no sex differences in the changes in cognitive flexibility (TAP 2.3 [66]), neither in reaction times nor in the error rate from before to after neurofeedback intervention.

Table 3 illustrates that both groups of adolescents with ASD (male/female) performed slightly better in emotion recognition as well as in cognitive flexibility (faster behavioral reactions, less errors) after the neurofeedback intervention, compared to before neurofeedback.

#### Psychological measures: co-occurring psychopathological symptoms of ASD

The analysis did not reveal any significant sex differences regarding treatment outcome measures on the psychological level. Neither, pre-post-intervention changes measured via self-report questionnaires (YSR), nor measured via parental reports (CBCL) yielded sex differences concerning the changes of co-occurring psychopathological symptoms.

The indices of treatment outcome (Δ delta values) in Table 4 illustrate that male adolescents with ASD experience greater improvements regarding externalizing problems, internalizing problems and total problems than female adolescents; however, these differences were not statistically significant (YSR). Although females with ASD reported a decrease of externalizing problems and total problems after the intervention, the internalizing score seemed to be unaffected or even very slightly increased after the intervention. Inspecting the data of the baseline measures Table 4 shows that male adolescents with ASD already reported slightly more externalizing and internalizing (as well as total) problems before the intervention than female adolescents.

**Table 4:** Treatment outcome (Δ delta index) of psychological measures

	Males (*n* = 6)			Females (*n* = 6) ^a^			Welch’s statistics	
	Pre-measurement, M (SD)	Post-measurement, M (SD)	Δ	Pre-measurement, M (SD)	Post-measurement, M (SD)	Δ	t (df)	*p*-value
*YSR/11-18R*								
Ext. problems	19.50 (7.66)	17.33 (7.34)	−2.17 (3.82)	16.00 (6.60)	14.40 (6.70)	−1.60 (2.07)	0.313 (7.926)	0.763
Int. problems	14.83 (12.51)	11.00 (7.00)	−3.83 (6.00)	14.20 (10.92)	14.80 (10.99)	0.60 (1.19)	1.69 (6.528)	0.139
Total YSR	72.17 (24.00)	60.33 (15.38)	−11.83 (13.59)	65.80 (23.68)	64.40 (30.28)	−1.40 (10.10)	1.46 (8.923)	0.179
*CBCL/6-18R*								
Ext. problems	14.83 (6.11)	15.00 (8.37)	0.17 (5.64)	19.5 (9.50)	15.17 (12.00)	−4.33 (6.50)	−1.28 (9.803)	0.230
Int. problems	13.83 (6.50)	7.83 (4.10)	−6.00 (4.15)	22.50 (6.32)	16.83 (6.91)	−5.67 (6.31)	0.11 (8.637)	0.916
Total CBCL	58.33 (14.26)	41.83 (17.56)	−16.50 (16.72)	78.67 (5.47)	56.17 (25.50)	−22.50 (21.44)	−0.54 (9.440)	0.601

*M* mean, *SD* standard deviation, *Ext. problems* externalizing problems, *Int. problems* internalizing problems, *TS* total score, *YSR* youth self report, *df* degrees of freedom, *CBCL* child behavior checklist

^a^For the YSR/11-18R, data from *n* = 5 female participants were included in statistical analysis

Examining the descriptive data of the parental ratings of co-occurring psychopathological symptoms (measured via CBCL), a contrary picture compared to adolescents’ self-ratings emerges. The caregivers of the female adolescents with ASD rated higher decreases in the CBCL total score after the neurofeedback intervention; however, the caregivers of the male adolescents with ASD evaluated the changes on the CBCL from pre to post differently than the caregivers of the females. Although they reported a decrease of internal and total problems after the neurofeedback intervention, externalizing problems did not change or even slightly increased after the intervention, as shown in Table 4.

## Discussion

The present study investigated sex differences regarding the effects of an intense neurofeedback training on emotion recognition abilities, cognitive flexibility and co-occurring psychopathological symptoms in male and female adolescents with ASD. A study including females with ASD seemed especially important as woman or girls are underrepresented in basic research and interventional treatment studies and specifically because of the underrepresentation in research in ASD.

Contrary to our expectations, analysis did not reveal any significant sex differences with respect to any treatment outcome measure used in our study. Neither delta values from pre to post regarding co-occurring psychopathological symptoms of ASD, nor delta values regarding cognitive flexibility or emotion recognition showed significant differences between male and female adolescents with ASD. Therefore, our findings do not correspond with a recent study about neurofeedback training on learning disorders [67] investigating 28 elementary school students with dyscalculia. Only in males could positive effects be evaluated after 20 sessions of beta/theta neurofeedback training.

Facing the different levels of measures, i.e. measures on the subjective psychological level (co-occurring psychopathological symptoms) versus measures on the behavioral level (emotion recognition, cognitive flexibility) of our study, an interesting picture emerged.

On one hand, the behavioral reactions in emotion recognition abilities are seemingly not influenced by the sex of the participant, with almost no measurable increase from pre to post for both male and female adolescents. Similarly, the behavioral data measuring cognitive flexibility also seem unrelated to the sex of the participants. Here, small improvements in the behavioral performances i.e. decreases in error rates and faster reaction times from pre to post could be registered for both sex, which is in line with the study of Kouijzer et al. [58]. Similar positive effects of neurofeedback on executive functions could be shown in children with ASD [31].

On the other hand, although not the pre-post-treatment change but the evaluation of co-occurring psychopathological symptoms of ASD seems to be related to the sex of the study participant. Significant sex differences were found at the time of premeasurement in the parental evaluations, as caregivers described significantly higher levels of burden (CBCL total score) for daughters than for sons with ASD, which is consistent with a study of Holtmann et al. [68].

Regarding the self-reported version of the same questionnaire (YSR), interestingly, the opposite picture emerged although this difference did not reach the level of significance (potentially due to the higher SD). Male adolescents with ASD rated their internalizing and externalizing problems on average as more severe than female adolescents with ASD.

Summing up on a descriptive level, it seems that boys with ASD rated their psychopathology as more severe than their caregivers, whereas the caregivers of girls with ASD rated the girls’ psychopathologies as more severe than the girls with ASD themselves, which partly contradicts previous research by Pisula et al. [69]. One possible explanation for the fact that girls assess their psychopathological symptoms as less severe than their caregivers could be explained by the camouflage effect [5, 6], which is in line with previous research showing that females tend to hide their difficulties in social contact [70]. Another explanation would be that there is a sex-based bias regarding caregivers’ expectations of their children as some authors described that caregivers generally expect higher levels of social interaction skills from daughters than from sons [71].

Regarding the tendency of co-occurring psychopathological symptoms of ASD to decrease after neurofeedback training, our results are consistent with previous studies. Similar to Mekkawy [59], reporting significant decreases in recurrent problem scores of the CBCL after neurofeedback training in children with ASD, our analysis showed decreases in the internalizing and externalizing scale as well as in the total scores of the CBCL after neurofeedback training.

Notwithstanding, the current study has several limitations and cannot provide generalizable results because of its experimental design and exploratory character. Besides the small sample size due to challenging recruitment of females with ASD and the emergence of COVID-19 restrictions, also possible other influences on the results, such the strong overlap of ASD with ADHD could not be ruled out in this study. As sex differences in ADHD were reported in many studies [24, 29, 30], disentangling and differentiating of the potentially influencing, underlying components would be an interesting area for future research. Furthermore, the investigation of possible age-related sex differences in neurofeedback training as well as examining potential sex differences regarding long-term effects of neurofeedback training in ASD may be other interesting future research ventures.

## Conclusion

The current study highlights the importance of measuring baseline data and treatment outcome on different levels, when aiming to investigate sex differences. Although the level of significance was not reached on the behavioral level as well as on the subjective psychological level, regarding changes in treatment outcome, the latter might potentially be more biased regarding sex differences in ASD. Especially, third party evaluators seem to rate the comorbid psychopathological symptoms differently related to the sex of the evaluated persons. If this is due to biased psychological rating scales (which do not portray the diverging sex-related clinical presentations of the disorder appropriately) or due to the already biased perspectives and expectations of the caregivers on characteristics and personal features of sex in general, is still an open question.

Nevertheless, the present study adds to the growing body of research on alternative and noninvasive treatment approaches for individuals with ASD, fulfilling the call [40] to close the gap of previous research and investigate potential sex differences in the field of neurofeedback therapy.

Considering the potential consequences of unnoticed sex differences in ASD treatment outcome research, our study sheds light on potential moderating factors on specific levels of assessment, which should be considered in future studies. In addition, the present study exemplifies the importance of recruiting female participants to overcome the dominance of studies with solely male subjects. Including larger sample sizes measured on different outcome levels would allow more accurate and specific interpretations and potential therapeutic adjustments related to the sex of the treated patient.
